# Guidelines on how to monitor gestational weight gain during antenatal care: Number 2 – February 2023

**DOI:** 10.1055/s-0043-1766109

**Published:** 2023-03-28

**Authors:** Fernanda Garanhani de Castro Surita, Renato Teixeira Souza, Thaís Rangel Bousquet Carrilho, Lilian de Paiva Rodrigues Hsu, Rosiane Mattar, Gilberto Kac

**Affiliations:** 1 Departamento de Tocoginecologia, Faculdade de Ciências Médicas, Universidade Estadual de Campinas, Campinas, SP, Brazil; 2 Departamento de Tocoginecologia, Faculdade de Ciências Médicas, Universidade Estadual de Campinas, Campinas, SP, Brazil; 3 Observatório de Epidemiologia Nutricional, Instituto de Nutrição Josué de Castro, Universidade Federal do Rio de Janeiro, Rio de Janeiro, RJ, Brazil; 4 Departamento de Ginecologia e Obstetrícia, Faculdade de Ciências Médicas, Santa Casa de São Paulo, São Paulo, SP, Brazil; 5 Departamento de Obstetrícia, Escola Paulista de Medicina, Universidade Federal de São Paulo, São Paulo, SP, Brazil; 6 Observatório de Epidemiologia Nutricional, Instituto de Nutrição Josué de Castro, Universidade Federal do Rio de Janeiro, Rio de Janeiro, RJ, Brazil

## Key points

- Gestational weight gain (GWG) should be tracked at all antenatal consultations, and pregnant women should gain weight according to pre-established guidelines. The recommendation on weight gain varies according to the pre-pregnancy nutritional status (body mass index – BMI).
- Pre-pregnancy weight can be obtained from medical records, reported by the pregnant woman, or measured at the beginning of pregnancy. Maternal height should be obtained at the beginning of pregnancy. The two measures are used to calculate the pre-pregnancy BMI and classify the nutritional status as underweight, normal weight/eutrophic, overweight or obesity.
- Until 2022, there was no weight gain curve for the Brazilian population and the Ministry of Health recommended the adoption of two tools for nutritional monitoring during pregnancy: Atalah’s gestational BMI curve (Chile) and GWG recommendations from the Institute of Medicine – IOM (United States).
- Based on data from 7,086 women participating in 21 studies that are part of the Brazilian Maternal and Child Nutrition Consortium (Portuguese acronym: CONMAI), specific GWG curves and recommendations were created for Brazilian pregnant women.
- The new GWG curves and recommendations were discussed and endorsed by specialists in a workshop held in Brasília in June 2020, and adopted by the Ministry of Health as of 3 August, 2022.
- The new system includes a single instrument that allows tracking and making recommendations on gestational weight gain.

## Recommendations

- In antenatal care, guidelines on optimal weight gain should be based on the new GWG recommendations for Brazilian pregnant women.
- Insufficient or excessive weight gain is associated with the occurrence of adverse maternal and neonatal outcomes.
- The use of specific weight gain curves for Brazilian women presented in this document is the current recommendation of national guidelines.
- Ranges of GWG according to pre-pregnancy BMI can also be used and should include the different guidelines for cumulative weight gain per trimester of pregnancy.
- Gestational weight gain is a modifiable factor, so it must be carefully tracked in order to reduce risks associated with weight gain out of the range.

## Background

Weight gain during pregnancy is natural and necessary for the growth and development of the fetus. Gestational weight gain (GWG) includes the following: fetus, placenta and amniotic fluid, uterus and breast growth, expansion of blood volume and extracellular fluid, and maternal body fat reserve. 1 2 Gestational weight gain tracking is an important part of the antenatal consultation and deserves attention from both health professionals and the pregnant woman.

Gestational weight gain out of recommended ranges is associated with maternal complications and adverse perinatal and infant outcomes. 2 3 Insufficient weight gain is associated with low birth weight, prematurity, birth of small for gestational-age (SGA) children, and neonatal mortality. 3 4 Excessive weight gain is also associated with adverse outcomes such as gestational diabetes, hypertensive pregnancy syndromes, postpartum weight retention (that may result in maternal obesity), prematurity, higher risk of undergoing cesarean delivery, birth of children with macrosomia or large for gestational age (LGA) and childhood obesity. 2 4

Since the early 2000s, the Ministry of Health has recommended the adoption of two methods for assessing the nutritional status of pregnant women during antenatal care: the Chilean curve by Atalah et al. 5 and recommendations of the Institute of Medicine (IOM) 2 from 2009. Guidance for health professionals on the diagnosis of the nutritional-gestational status included the adoption of the curve and table of body mass index (BMI) by gestational age by Atalah et al. 5 and of GWG recommendations according to the IOM. 5 6 Although the curve by Atalah et al. 5 did not depend on pre-pregnancy weight information and early initiation of antenatal care, it had several important methodological problems, such as the use of outdated cutoff points for classifying nutritional status at the beginning of pregnancy, in disagreement with recommendations of the World Health Organization (WHO) since 1995. 5 7 In this curve, women with excessive weight gain were also classified as adequate, which could contribute to the overweight and obesity epidemic observed among Brazilian women in recent decades. 8

Although there is no global recommendation on ideal GWG values, since the 1990s, the recommendations proposed for certain countries consider different weight gain ranges according to the pre-pregnancy BMI. 9 The most used recommendations worldwide are those developed by the IOM in the United States in 2009, adopted in full or in part in different countries. 2 10 These were developed for North American pregnant women, considering the epidemiological scenario at the time and the reality of high-income countries.

Given the aforementioned limitations, these tools have to be updated considering different populations and cultures, as well as the local epidemiological scenario. Parameters also have to be established so that health professionals can guide pregnant women. Between 2021 and 2022, Brazilian researchers developed specific GWG curves and recommendations for the Brazilian population, adopted by the Ministry of Health from 2022 onwards. 11 12 These new curves and recommendations should replace current conduct and allow that health professionals provide clear guidelines from the beginning of pregnancy, aiming at maintaining adequate weight gain and reducing the risk of adverse maternal and child outcomes.

## Why create GWG curves for Brazilian pregnant women?

To avoid errors arising from the use of curves based on anthropometric data and perinatal outcomes from other populations, Brazilian GWG curves were created based on data from the Brazilian Maternal and Child Nutrition Consortium (Portuguese acronym: CONMAI), a collaborative research network created in 2019 to investigate issues of maternal and child health and nutrition. 13 These curves were created using data from apparently healthy Brazilian women who participated in studies conducted in Brazil between 1990 and 2018, and published in 2021. 11 Four cumulative GWG curves were created according to the pre-pregnancy BMI category. These curves allow the evaluation of weight gain at each antenatal visit, depending only on weight measurement at the visit in question and a pre-pregnancy weight measurement. During the process of preparing the curves, the group of researchers from CONMAI also demonstrated the possibility of using self-reported pre-pregnancy weight to calculate BMI and GWG. 14

## How were the GWG recommendations defined?

The GWG curves represent an important advance for monitoring the behavior of this indicator in Brazil. However, they do not answer the most common question asked by pregnant women during antenatal care: what is the recommended weight gain until the end of pregnancy? The percentiles on curves associated with higher/lower risk of adverse outcomes also needed to be identified. Thus, optimal GWG ranges were defined based on values that would reduce the occurrence of births of SGA and LGA children, premature children and excessive weight retention at 6 and 12 months postpartum. Data from CONMAI and the Food and Nutrition Surveillance System were used in this analysis. Details on data and methods used in the definition of these ranges will be published soon. 12

**Figure 1.**
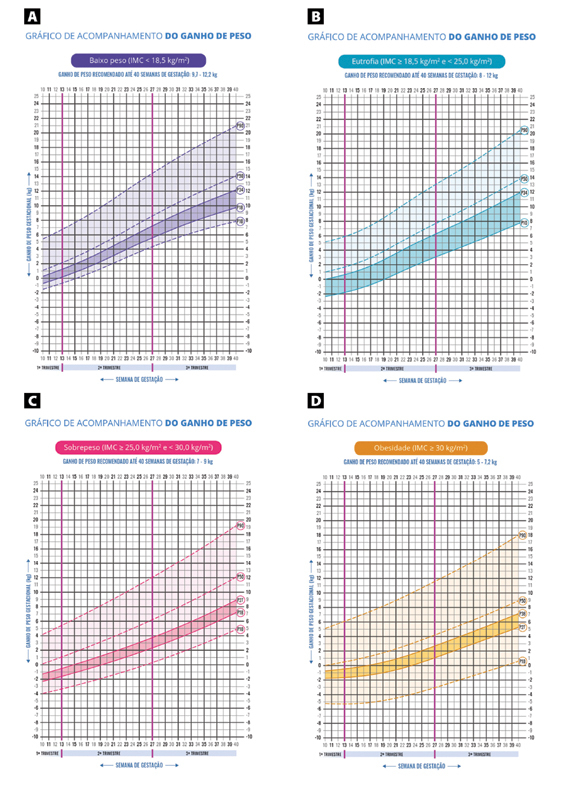
Gestational weight gain curves for Brazilian women according to pre-gestational body mass index (BMI). **Source:** Adapted from Ministry of Health (2022). 15 (A) underweight pregnant women (BMI < 18.5 kg/m ^2^ ); (B) eutrophic pregnant women (>18.5 and <25.0 kg/m ^2^ ); (C) overweight pregnant women (≥25 and <30 kg/m ^2^ ); (D) obese pregnant women (≥30 kg/m ^2^ ).

The optimal GWG ranges were proposed by researchers from the Nutritional Epidemiology Observatory of the Universidade Federal do Rio de Janeiro (UFRJ) and discussed with technicians from the Ministry of Health, health professionals who perform antenatal care and specialist researchers from various regions of Brazil in a workshop held in June 2022, in Brasília. The new guidelines agreed upon in this workshop were incorporated into the curves and into the new pregnant woman’s record card ( Figure 1 ) ( Chart 1 ). Therefore, the diagnosis of anthropometric nutritional status during pregnancy, monitoring and guidance on GWG can be performed using a single instrument.

**Chart 1.** Gestational weight gain recommendation ranges according to pre-pregnancy body mass index (BMI)

Pre-pregnancy BMI (kg/m ^2^ ) 7	Classification of pre-pregnancy BMI*	Adequacy range in the chart (percentile) 12	Cumulative weight gain (kg) per trimester**		
			Up to 13 weeks (1 ^st^ trimester)	Up to 27 weeks (2 ^nd^ trimester)	Up to 40 weeks (3 ^rd^ trimester)
<18.5	Underweight	P18-P34	0.2 - 1.2	5.6 - 7.2	9.7 - 12.2
≥18.5 and <25	Normal weight	P10-P34	-1.8 - 0.7	3.1 - 6.3	8.0 - 12.0
≥25 and <30	Overweight	P18-P27	−1.6 - −0.05	2.3 - 3.7	7.0 - 9.0
≥30	Obesity	P27-P38	−1.6 - −0.05	1.1 - 2.7	5.0 - 7.2

**Note:** Up to 13 weeks of pregnancy, a small weight gain is expected for underweight (up to 1.2 kg) and normal weight women (0.7 kg). No weight gain is expected for overweight or obese women. For eutrophic, overweight and obese women, a small weight loss (maximum 1.5 kg) may occur.

**Source:** World Health Organization (1995) 7 and Carrilho et al. (2022). 12

The GWG curves and guidelines were created for adult pregnant women (over 18 years old) in low-risk singleton pregnancies. There are still no specific guidelines for different degrees of obesity. The recommendation ranges were defined based on the best evidence available to date and considering the current epidemiological scenario. This scenario needs to be monitored and the ranges may be revised in the future.

## How to use the curves and guidelines in clinical practice?

Health professionals who will monitor the pregnant woman and advise on GWG should adopt the following guidelines:

1. In the first consultation, the pre-pregnancy BMI should be calculated using the formula pre-pregnancy BMI = pre-pregnancy weight (kg)/height (m) ^2^ . The pre-pregnancy weight used in this calculation must be the one reported by the pregnant woman. When the pregnant woman cannot inform this value, the weight measured at the beginning of pregnancy (up to 8 weeks) or the usual weight of the pregnant woman should be considered.
2. The pre-pregnancy BMI must be classified according to cutoff points proposed by the WHO (WHO, 1995) 7 and presented in chart 1. The adequate tracking curve must be chosen (Figure 1) from this classification.
3. After obtaining the weight at the consultation, the GWG (kg) must be calculated using the formula: GWG = weight at the visit – pre-pregnancy weight. This value must be marked on the chosen curve (gain value per gestational age). From the marking, it is possible to assess if the gain is within recommended ranges (darker areas on the graph) or above/below recommendations.
4. The professional should advise the pregnant woman on maintaining adequate weight gain or on the need to increase/reduce gain, so that she reaches the recommended values.
5. The weight gain schedule until the next appointment, until the end of the trimester or up to 40 weeks can also be calculated.

## Tools available for the use of curves by health professionals and pregnant women

Three tools were created to facilitate the adoption of the new curves and guidelines by health professionals performing antenatal care and pregnant women:

1. An interactive panel available in English and Portuguese that allows the use of curves and guidelines online (https://observatorioufrj.shinyapps.io/GPG_app/);
2. An Excel calculator that allows calculation of the z score and exact percentile of a pregnant woman or a database, including multiple measurements by pregnant women and several pregnant women (https://dataverse.nutricao.ufrj.br/dataverse/curvas_openaccess/);
3. An Android/iOS application (PesoGestBR) including curves, recommendations and nutritional guidelines based on the Food Guide for the Brazilian Population (Brazil, 2014). Although created specifically for pregnant women, it can be used by health professionals, as the registration of more than one pregnant woman is allowed. This app will be available for free download until December 2022.

## Final considerations

Following the proposed recommendations for weight gain during pregnancy is important for the woman and for the outcome of the pregnancy. Thus, the curves and recommendations should be widely disseminated and used by health professionals. Following recommendations and adapting weight gain is not an easy task at any stage of life, and it is no different during pregnancy. Pregnancy brings several physical and emotional demands that must be considered by the team of professionals performing antenatal care. The pregnant woman needs to be served with cordiality and without judgments or stigmas because of her nutritional condition. This way, she will be more likely to monitor her weight gain, adhere to recommendations and reduce the risk of maternal and child adverse outcomes.

National Commission Specialized in Antenatal Care of the Brazilian Federation of Gynecology and Obstetrics Associations (Febrasgo)

President:

Fernanda Garanhani de Castro Surita

Vice-president:

Lílian de Paiva Rodrigues Hsu

Secretary:

Adriana Gomes Luz

Members:

Eliana Martorano Amaral

Eugenia Glaucy Moura Ferreira

Francisco Herlanio Costa Carvalho

Joeline Maria Cleto Cerqueira

Jorge Oliveira Vaz

Jose Meirelles Filho

Luciana Silva dos Anjos França

Marianna Facchinetti Brock

Mary Uchiyama Nakamura

Patricia Gonçalves Teixeira

Renato Ajeje

Sergio Hecker Luz

National Commission Specialized in High Risk Pregnancy of the Brazilian Federation of Gynecology and Obstetrics Associations (Febrasgo)

President:

Rosiane Mattar

Vice-president:

Alberto Carlos Moreno Zaconeta

Secretary:

Mylene Martins Lavado

Members:

Arlley Cleverson Belo da Silva

Carlos Alberto Maganha

Elton Carlos Ferreira

Felipe Favorette Campanharo

Inessa Beraldo de Andrade Bonomi

Janete Vettorazzi

Maria Rita de Figueiredo Lemos Bortolotto

Fernanda Santos Grossi

Renato Teixeira Souza

Sara Toassa Gomes Solha

Vera Therezinha Medeiros Borges
